# Enhanced Detection of Low-Abundance Human Plasma Proteins by Integrating Polyethylene Glycol Fractionation and Immunoaffinity Depletion

**DOI:** 10.1371/journal.pone.0166306

**Published:** 2016-11-10

**Authors:** Zhao Liu, Songhua Fan, Haipeng Liu, Jia Yu, Rui Qiao, Mi Zhou, Yongtao Yang, Jian Zhou, Peng Xie

**Affiliations:** 1 Department of Neurology, The First Affiliated Hospital of Chongqing Medical University, Chongqing, China; 2 Institute of Neuroscience and the Collaborative Innovation Center for Brain Science, Chongqing Medical University, Chongqing, China; 3 Chongqing Key Laboratory of Neurobiology, Chongqing, China; 4 Department of Neurology, Yongchuan Hospital, Chongqing Medical University, Chongqing, China; Shantou University Medical College, CHINA

## Abstract

The enormous depth complexity of the human plasma proteome poses a significant challenge for current mass spectrometry-based proteomic technologies in terms of detecting low-level proteins in plasma, which is essential for successful biomarker discovery efforts. Typically, a single-step analytical approach cannot reduce this intrinsic complexity. Current simplex immunodepletion techniques offer limited capacity for detecting low-abundance proteins, and integrated strategies are thus desirable. In this respect, we developed an improved strategy for analyzing the human plasma proteome by integrating polyethylene glycol (PEG) fractionation with immunoaffinity depletion. PEG fractionation of plasma proteins is simple, rapid, efficient, and compatible with a downstream immunodepletion step. Compared with immunodepletion alone, our integrated strategy substantially improved the proteome coverage afforded by PEG fractionation. Coupling this new protocol with liquid chromatography-tandem mass spectrometry, 135 proteins with reported normal concentrations below 100 ng/mL were confidently identified as common low-abundance proteins. A side-by-side comparison indicated that our integrated strategy was increased by average 43.0% in the identification rate of low-abundance proteins, relying on an average 65.8% increase of the corresponding unique peptides. Further investigation demonstrated that this combined strategy could effectively alleviate the signal-suppressive effects of the major high-abundance proteins by affinity depletion, especially with moderate-abundance proteins after incorporating PEG fractionation, thereby greatly enhancing the detection of low-abundance proteins. In sum, the newly developed strategy of incorporating PEG fractionation to immunodepletion methods can potentially aid in the discovery of plasma biomarkers of therapeutic and clinical interest.

## Introduction

Recently, the discovery of reliable disease biomarkers is one of the biggest concerns for researchers [1–5]. Human blood plasma is one of the most studied biological fluids and is the main sample type used for disease diagnosis [6–8]. In contrast to brain tissue and cerebrospinal fluid, human plasma can be sampled easily without invasive procedures. Importantly, it contains various proteins that are actively secreted or presented following cell and tissue leakage [3]. These proteins are involved in protein transport, immune defense mechanisms, coagulation, and protease inhibition, and their levels can provide an indication of an individual’s physiological or pathological states [9–11].

Mass spectrometry (MS)-based plasma proteomics is a useful means for identifying novel clinical biomarkers that can provide unambiguous protein assignments [12]. However, because of the huge complexity and different concentrations of component proteins, the analysis of plasma proteome faces the great challenges [3]. Moreover, the detection limits of MS imposed by the various ionization processes employed impact both the complexity and dynamic range of analytes that are measurable [13]. These disadvantages are reflected by the predominance of high- and moderate-abundance proteins (HAPs and MAPs), which clearly hampers the identification and quantification of potential low-abundance protein (LAP) biomarkers [2]. Consequently, the LAPs among a large excess of other proteins, detected by MS is key for studying particular diseases both with respect to the limited amount of protein analyzed and to the given quantity of certain proteins in the sample [14]. During the last few decades, the most commonly used approach to facilitate LAP analysis has been to reduce plasma sample complexities by fractionation [15].

Substantial effort has been directed at improving fractionation strategies with human plasma samples, and many different techniques have been developed and applied [16]. A current popular approach is immunoaffinity depletion (IAD), in which antibodies are used to capture the most abundant proteins [17]. Immunodepletion can facilitate the analysis of the next tier of proteins by eliminating some of the most abundant proteins. This effective strategy is increasingly being applied in various biomarker studies [1, 18]. Alternatively, protein ultrafiltration and precipitation strategies, discriminated by size or size/pI, respectively, have also been widely applied prior to sample profiling by electrophoresis or MS [19, 20]. Centrifugal ultrafiltration is used to for enrich low-molecular-weight proteins, which are considered as an important source of biomarkers [21, 22]. In contrast, protein precipitation is often more preferable other than ultrafiltration, because the membranes are not so robust enough for high protein concentrations and suffering from fouling that used for ultrafiltration [23, 24]. Recent reviews have systematically described the increasing number of initial fractionation approaches used for plasma proteomic analysis [25]. Unfortunately, current single-step analytical approaches cannot reduce the intrinsic complexity of plasma proteome and only identify a limited number of proteins. However, various multi-step processes can identify a high number of proteins with confidence [26, 27].

Because the chance of discovering new possible biomarkers increases with the number of proteins profiled, the aim of this study was to integrate known approaches into a relatively simple and rapid method for comprehensively profiling the human plasma proteome. Accordingly, we present a combined strategy for analyzing the human plasma proteome by integrating polyethylene glycol (PEG) fractionation and immunoaffinity depletion. This integrated system was applied to a liquid chromatography (LC)-MS/MS-based plasma proteome. Herein, we present comparative results to illustrate the potential capability of our newly developed strategy for enhancing the detection of LAPs in plasma.

## Materials and Methods

### Human blood plasma sample

The protocols of this study were reviewed and approved by the Ethical Committee of Chongqing Medical University. The methods were carried out in accordance with the approved guidelines and regulations. From 10/12/2012 to 11/13/2012, all human blood samples were collected at the medical examination center of the First Affiliated Hospital of Chongqing Medical University from the healthy individuals after written informed consent. Blood was harvested in collection tubes with ethylenediaminetetraacetic acid from 6 healthy individuals (3 male and 3 female) aged 18–50 yrs. Then, the plasma samples were prepared as described in our previous study [1] and pooled in equal volumes. The initial protein concentration was approximately 66.6 mg/mL as determined by using the Bicinchoninic Acid (BCA) Protein Assay Kit, according to the manufacturer’s instructions (Pierce, USA).

### PEG fractionation (PEGF)

**Fig 1:**
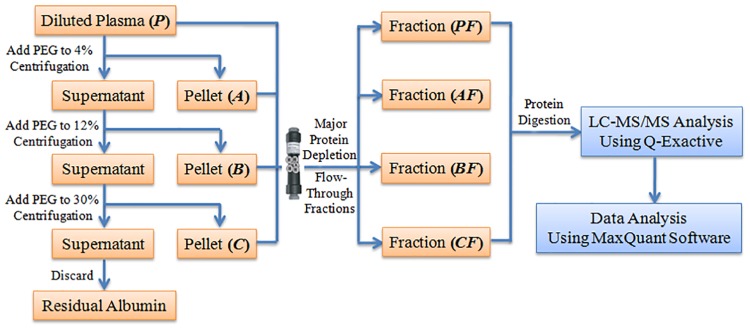
Schematic representation of the combination of differential PEG precipitation and immunoaffinity chromatography. Diluted human plasma was fractionated by PEG precipitation followed by IAD of seven major high-abundant proteins. The obtained flow-through fractions were subjected to in-solution tryptic digestion, and the resultant peptides were analyzed by LC-MS/MS. The obtained data was analyzed using MaxQuant software.

An initial fractionation of whole-plasma human proteins based on PEG precipitation is shown in Fig 1. Typically, 30 μL of plasma (~2 mg proteins) was diluted in phosphate-buffered saline (pH 7.2–7.4) to 500 μL. To the diluted sample, a 48% (w/v) PEG 6000 stock solution was added to a final concentration of 4% PEG. The PEG-suspended solution was placed on ice for 30 min to ensure protein precipitation and then centrifuged at 1500 × *g* at 4°C for 10 min. The resulting pellet was designated as fraction A. The supernatant was further treated similarly as described for fraction A to produce 12% and 30% PEG precipitation fractions, which are designated as the fraction B and fraction C, respectively. However, the centrifugation steps for fractions B and C were performed at 12,000 × *g* for 15 min. The final supernatant from the 30% PEG precipitation was collected and then extracted with ice-cold acetone containing 0.07% (v/v) β-mercaptoethanol. All obtained fractions were stored at -80°C until further analysis.

### Immunodepletion of HAPs from plasma

As depicted in Fig 1, the A, B, and C fractions as obtained above were re-dissolved in “Buffer A” (Agilent Technologies), and the non-fractioned reference plasma sample (P) was diluted. After centrifugation, these protein samples were immunodepleted of seven plasma HAPs (albumin, IgG, antitrypsin, IgA, transferrin, haptoglobin, and fibrinogen) using a Multiple Affinity Removal Column Human-7 (4.6 mm inside diameter [ID]×50 mm; Agilent Technologies, USA) [1], according to the manufacturer’s instructions. “Buffer B” (Agilent Technologies) was used to elute the bound proteins. The flow-through fractions (LAPs and MAPs) were collected, desalted, and concentrated with 5-kDa Molecular Weight Cutoff Centrifugal filters (Amicon Ultra, Millipore) to generate the corresponding AF, BF, CF, and PF samples.

### One-dimensional denaturing electrophoresis

The samples were mixed with a denaturing buffer containing 125 mM Tris-HCl, pH 6.8, 1% SDS (w/v), 50 mM dithiothreitol (DTT), 10% glycerol (v/v), and 0.001% bromophenol blue (w/v), and was then boiled in water for 5 min in preparation for SDS-PAGE analysis [28]. After centrifugation at 14,000 × *g* for 15 min, the proteins in the supernatants were resolved in parallel lanes using a 4.8% stacking gel and a 10% separation gel and then stained with Coomassie Blue R-250.

### One-dimensional non-denaturing electrophoresis

Non-denaturing electrophoresis was performed according to Sun et al. [29]. The samples were re-dissolved directly in non-denaturing buffer containing 125 mM Tris-HCl, pH 6.8, 10% glycerol (v/v), and 0.001% bromophenol blue (w/v). A vertical discontinuous gel system consisting of 7.5% separating and 4.0% stacking gels was employed. Electrophoresis buffer was prepared by dissolving 3.0 g Tris and 14.4 g glycine in Milli-Q water and adjusting the pH to 8.3. The voltage in the stacking gel was set to 120 V. After ~30 min, when the sample entered the separating gel, the voltage was turned down to 90 V and kept voltage constant until the bromophenol blue indicator moved to the bottom of the gels.

### Two-dimensional denaturing electrophoresis (2DE)

The samples were mixed with a rehydration solution containing 7 M urea, 2 M thiourea, 4% CHAPS, 50 mM DTT, 0.2% Bio-Lyte, and 0.001% bromophenol blue(w/v) to a total volume of 350 μL, and then used to rehydrate 17 cm-long pH 3–10 NL, pH 4–7 L, and pH 5–8 L IPG strips. The strips were passively rehydrated for 16 h in 350 μL of sample at 25°C, after which 1 mL of mineral oil was added. A multi-step IEF voltage program was applied to the strips on a Protean IEF cell: 50 V for 12 h, 250 V for 30 min, 1000 V for 1 h, 1000 V to 10,000 V over a 5 h step-up period, and 10,000 V for 6 h. The strips were equilibrated in reduction buffer (0.375 M Tris-HCl, pH 8.8, 6 M urea, 20% glycerol [v/v], 2% SDS [w/v], and 2% DTT [w/v]) and then in the same buffer containing 2.5% IAA instead of 2% DTT. The second dimension of electrophoresis was accomplished by running the strips on 1mm-thick 10% SDS-polyacrylamide vertical slab gels using a Protean^®^ Π xi Multi-Cell (Bio-Rad). Protein condensation and separation were performed at 12.5 mA/gel for 30 min and 25 mA/gel for 5.0–5.5 h at 20°C. Protein spots were visualized by silver staining and scanned using an Epson 10000XL scanner, according to our previous studies [30, 31].

### In-solution protein digestion

Protein pellets were dissolved in SDT buffer (4% SDS, 10 mM DTT, and 150 mM Tris-HCl, pH 8.0), and the resulting protein solutions were further diluted in SDT buffer. The lysates were boiled in water for 5 min, and then centrifuged at 40,000 × *g* for 15 min. Protein concentrations were determined using the BCA Protein Assay Kit (Pierce, USA). Bovine serum albumin was used as a standard. Subsequent protein digestion was performed using the filter-aided sample preparation method in a 5-kDa Molecular Weight Cut-Off Centrifugation Filter (Sartorius) [32, 33]. The sample was diluted with 200 μL of UA buffer (8 M urea, 150 mM Tris-HCl, pH 8.0) and centrifuged at 14,000 × *g* for 30 min. This step was repeated once. Subsequently, 100 μL of 50 mM iodoacetamide in UA buffer was added to the filters and the samples were incubated in darkness for 30 min. Filters were washed twice with 100 μL of UA buffer, followed by two washes with 100 μL of 25 mM NH_4_HCO_3_. Proteins were digested overnight in 40 μL 25 mM NH_4_HCO_3_ (pH 8.5) using trypsin at a trypsin-to-protein ratio of 1:50 at 37°C. The released peptides were collected by centrifugation at 14,000 × *g* for 10 min followed by 2 washes with 25 mM NH_4_HCO_3_, and their concentrations were measured by determining optical density values at 280 nm.

### LC-MS/MS

Two micrograms of tryptic peptides prepared as described above were used for LC-MS/MS analysis, after loading on a reversed-phase C18 trap column (EASY Column SC001, 20 mm×150 μm ID). Reversed-phase chromatography was performed using a Thermo EASY-nLC 1000 Column (Thermo Finnigan) with a binary buffer system consisting of 0.1% formic acid (buffer A) and 84% acetonitrile in 0.1% formic acid (buffer B). A reversed-phase C18 analysis column (EASY Column SC200, 100 mm×150 μm ID) was used. Online LC separation was performed using a 0–45% buffer B gradient over 100 min, a 45–100% buffer B gradient over 8min, followed by 100% buffer B for 12 min. The flow rate was 400 nL/min. The column was operated at a constant temperature of 35°C, which was regulated by an in-house-designed oven with a Peltier element. Peptides eluted from the LC column were directly injected into a coupled Q-Exactive mass spectrometer (Thermo Finnigan) via an anoelectrospray source (Proxeon Biosystems; now Thermo Fisher Scientific). The Q-Exactive instrument was operated in the data-dependent mode with survey scans acquired at a resolution of 70,000 at m/z 200 (transient time, 256 ms). Up to the top ten most abundant isotope patterns with charges ≥2 from the survey scan were selected with an isolation window of 1.6 Th and fragmented by higher-energy collisional dissociation (HCD) with normalized collision energies of 25. The maximum ion injection times for the survey scans and MS/MS scans were 20 and 60 ms, respectively, and the ion target value for both scan modes was set to 10^6^. In this mode of operation, MS/MS scans were acquired with maximum speed because filling of the HCD trap occurred almost completely in parallel with acquisition of the transient from the preceding scan. Furthermore, because virtually all scans “time out” at 60 ms due to high target values, the maximum ion signal in the fragmentation spectra was obtained.

### Data analysis

Raw MS files were analyzed using the MaxQuant software environment (version 1.3.0.5). The MS/MS spectra were searched using the Andromeda search engine against a decoy version of Uniprot Human database (release 2013_03, 133,549 protein sequences). The database also included common contaminants. MaxQuant analysis included an initial search with a precursor mass tolerance of 20 ppm, a main search-precursor mass tolerance of 6 ppm, and a fragment mass tolerance of 20 ppm. The search included enzymes such as trypsin, variable modifications due to methionine oxidation and N-terminal acetylation, and fixed modifications of carbamidomethyl cysteine. The minimum peptide length was set to seven amino acids, and the maximum number of missed cleavages was set to two. The false-discovery rate (FDR) was set to 1% during both peptide and protein identification. The proteins identified by the same set of peptides were grouped and recorded as one protein group. The “match between runs” function was used, and the time window was set as 2min. The protein and peptide tables were filtered to remove protein identifications from the reverse database and common contaminants. The results reported here in bold red represent the proteins identified with at least one unique peptide, and the ion score cutoff was set to 18. To avoid apparent misidentifications resulting from protein name discrepancies [34], we manually examined the gene names and UniProt IDs and compared them to the list of proteins provided by the manufacturer.

Furthermore, the intensity-based absolute quantification (iBAQ) option of MaxQuant was used to calculate, based on the sum of peak intensities, the approximate abundance of each protein in the measured sample [35]. The compute pI/MW tool (http://web.expasy.org) was employed to calculate the theoretical molecular weights (MWs) and isoelectric points (pIs) of the identified proteins.

## Results

### Effective fractionation of human plasma proteins using PEG

**Fig 2:**
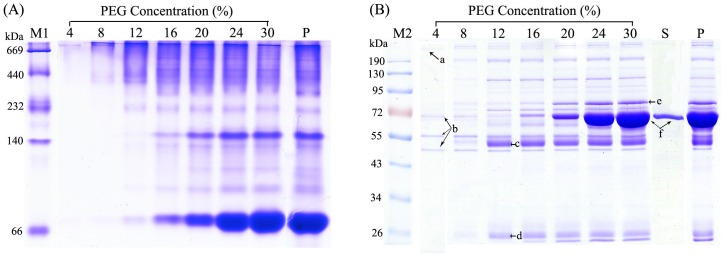
One-dimensional non-denaturing (A) and denaturing (B) electrophoretic analysis of plasma proteins following differential PEG fractionation steps, or no PEG fractionation step. The PEG concentrations used for precipitation are indicated above each lane, lane “S” represents the final supernatant from the 30% PEG precipitation that was collected following further treatment with cold acetone, and lane “P” shows results with a reference plasma sample. The molecular weight markers (M1, M2) are shown on the left of the images. Prior to electrophoresis analysis, all resulting pellets were re-dissolved with non-denaturing or denaturing buffer, as described in the Methods section. a: Fn; b: α, β, and γ-chain of Fb; c: IgG heavy chain; d: IgG light chain; e: transferrin; f: albumin.Unlike the majority of protein-fractionation agents, PEG separates protein components from natural mixtures by an exclusion mechanism [36]. Atha et al. analyzed the mechanism of PEG precipitated proteins and concluded that PEG, regard as an inert solvent sponge, can discretionarily raise the effective concentration of all proteins, and the proteins of larger size are more sensitive than the smaller proteins [37]. As expected, our non-denaturing electrophoresis results showed that some relatively high-MW proteins were first precipitated at a low PEG concentration (Fig 2A). Because the precipitation procedures were performed at low temperature, the component types in the obtained fractions might represent native protein complexes. To some extent, such a phenomenon has been observed and supported by the initial analysis with non-denaturing electrophoresis. For example, a 4% PEG precipitate was reported to contain fibronectin (Fn)-fibrinogen (Fb) complexes [38]. This phenomenon implied that attributable to the non-ionic and non-denaturing nature of PEG, the proteins fractionated by PEG could recover its native form, and could be used in subsequent non-denaturing immune affinity chromatography.

In the present study, human plasma was used as a source of proteins, and the whole-plasma proteins were precipitated with using PEG at various concentrations (4, 8, 12, 16, 20, 24, and 30%). To preliminarily evaluate the ability of PEG to fractionate plasma proteins, each of the resulting precipitates, together with non-fractioned reference plasma (P) and the final supernatant (S) from the 30%-PEG precipitation, were separately resolved by one-dimensional non-denaturing (Fig 2A) and denaturing gel electrophoresis (Fig 2B).

We also examined the PEG concentration used to precipitate human plasma proteins by one-dimensional denaturing electrophoresis. We expect that the fractionation procedure described enables the primary separation of known major plasma protein components into individual fractions and facilitates the study of minor components. As shown in Fig 2B, it was observed that the 4% PEG precipitate contained 4 main electrophoretic bands. According to the MW distribution observed here and in previous studies, these bands may represent known plasma proteins (i.e., high-molecular weight Fn and 3 different chains [α, β, and γ] of Fb) [38]. After increasing the PEG concentration to 12%, the high-abundance IgG heavy and light chain proteins were precipitated. Subsequently, the major HAP albumin began to gradually precipitate. In general, 30% is considered the maximal PEG concentration required to precipitate almost all proteins in solution. Electrophoretic analysis showed that the 30% PEG precipitate potentially contained a large percentage of albumin, whereas the final supernatant had only a small amount of this protein, and therefore was not considered to contain the low-abundance proteome.

### Effect of PEGF on distribution of immunodepleted plasma proteins

**Fig 3:**
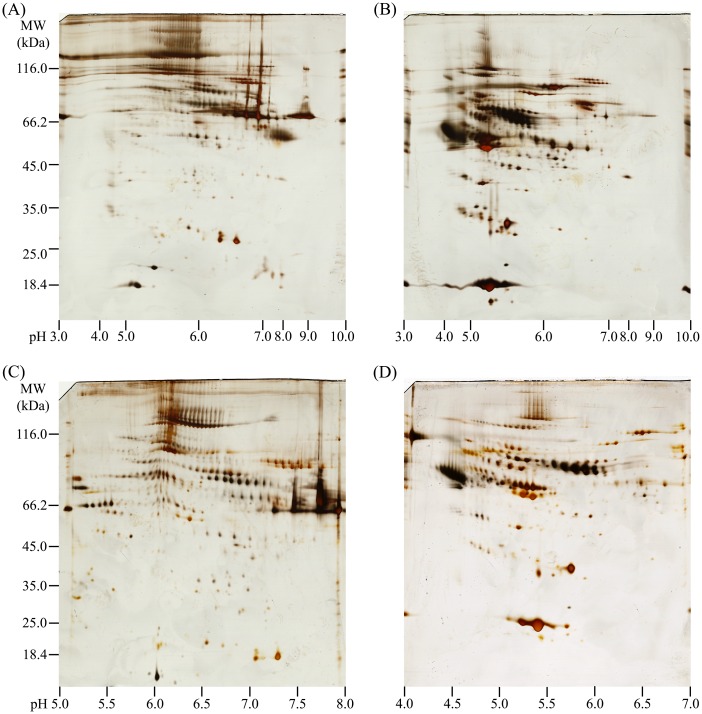
Two-dimensional gel images of protein mixtures from the BF (A, C) and CF (B, D) obtained following the PEGF-IAD method. Analysis was performed on pH 3–10 NL (A, B), pH 5–8 L (C), and pH 4–7 L (D) IPG strips in the first dimension and 12% SDS gels in the second dimension.To gain comprehensive insight into the bias characteristics of proteins from the BF and CF samples, we further employed an iBAQ-based proteomics approach. In the present study, three independent experiments were performed for technical replicates of the overall PEGF-IAD operational process. To rank the absolute abundance of different proteins within a single sample, we used the iBAQ algorithm. Although this algorithm is not quantitative because the two fractionated samples were markedly different in terms of protein compositions, this estimation still facilitated the identification of relative protein abundances. The computed iBAQ values were plotted as a function of either the theoretical MW or pI values for each identified protein. As shown in Fig 4, the proteins with high iBAQ values tended to show high MWs in the BF and moderate MWs in the CF. In terms of pI, the proteins with high iBAQ values mainly distributed in a pI range of 6–8 in the BF and 5–7 in the CF. We also employed the corresponding reciprocals of the iBAQ values to probe the distributions of LAPs in the BF and CF. As shown in S1 Fig, it was observed that more LAPs with high MW values were identified in the BF than in the CF.

Conventional 2DE permits the separation of protein samples according to their pI and MW [39], and was used herein to initially analyze the physicochemical characteristics of the AF, BF, and CF. The 2DE analyses were performed twice and the representative images were shown in Fig 3A–3D. The overall protein compositions are displayed in a silver-stained gel using a broad-range IPG strip (pH 3–10) for the IEF step. In the AF image, we did not observe any protein spots visually (data not shown), which may have been due in part to the sample characteristics and the low detection limit of 2DE. Shotgun analysis of this fraction led to the identification of over 300 proteins, which is likely attributable to the higher sensitivity of MS detection (S1 Table). In the BF image, most protein spots were spread over a relatively high MW range (>45.0 kDa) and a pI range of 5–8, while the protein spots in the CF image tended to reside within a pI range of 4–7. In light of these findings, we further selected a narrow range for the BF (IPG 5–8) and CF (IPG 4–7) samples. We observed that more distinguishable spots were displayed in the resultant images, compared to that observed with the IPG 3–10 strips (Fig 3C and 3D). These results suggested that 2DE analysis of the BF and CF samples based on narrower-range IPG strips might provide improved gel patterns in terms of resolution, number of spots, and minimal streaking. Thus, the use of narrow-range IPG strips in combination with PEGF should be helpful for gel-based analysis of plasma proteome.

**Fig 4:**
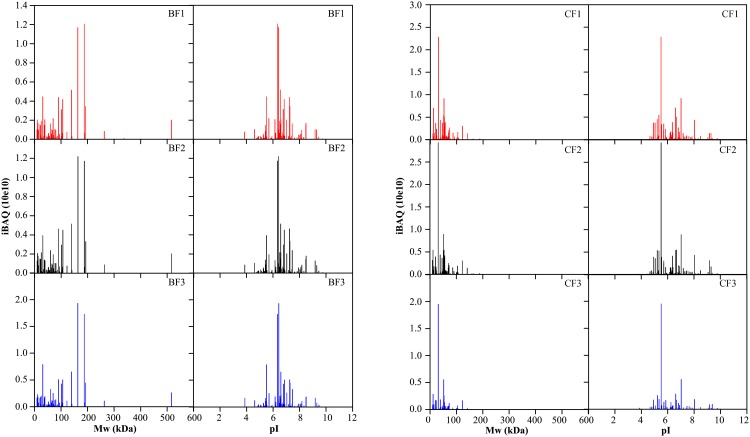
Plots of MW and pI versus iBAQ values of the plasma proteins identified from the BF and CF. In the two fractions, values for each replicate are plotted separately to illustrate consistency in the overall trends.

Indeed, the protein-distribution trends observed in the BF and CF were mainly limited to those comprising large, visible spots in the gels, most of which might be corresponding to MAPs except for the depleted HAPs. Adversely, some LAP spots may be difficult to visualize in 2DE gel images. These considerations may explain the fact that many LAPs in the BF tended to show low pIs (S1 Fig), whereas the corresponding gel showed a different tendency (Fig 3). Moreover, some proteins that clustered together on the gel were visible as chains of spots, but represented the same proteins. These proteins presumably showed altered running characteristics due to potential post-translational modifications and occurred as highly similar isoforms. Taken together, the characteristic trends of the BF and CF found by 2DE to some degree resembled those observed with the iBAQ-based method.

### Enhanced proteome coverage and LAP identification by the integrated strategy

**Fig 5:**
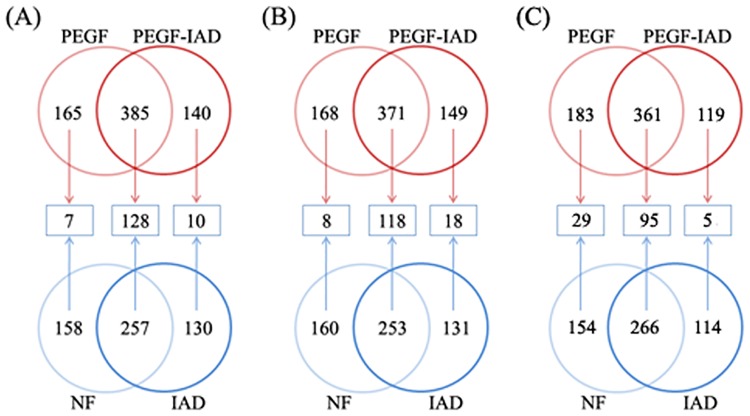
Evaluation of effect of combining the PEGF procedure with IAD-based characterization of the human plasma proteome in three independent experiments. In panels A, B and C, the values for each replicate are plotted separately to illustrate consistency in the overall trends. NF, non-fractionation.

To initially evaluate the effect of PEGF on the IAD-based identification of plasma proteins, the data of total proteins identified from PEGF and non-fractioned plasma were comparatively analyzed before and after IAD. The Venn diagram in Fig 5 shows the number of shared and unique proteins obtained in the three independent analyses. We observed an increased identification rate that mainly reflected the number of proteins identified in common between both methods, which might be substantially attributed to PEGF in terms of improving the efficiency of the IAD-based plasma proteome. The IAD method does not result in an increased number of identified proteins compared to non-depleted plasma; however, inclusion of the PEGF step can lead to a 30.9±1.5% (mean ± SD, similarly hereinafter) increase compared to that observed with non-fractionated plasma.

**Fig 6:**
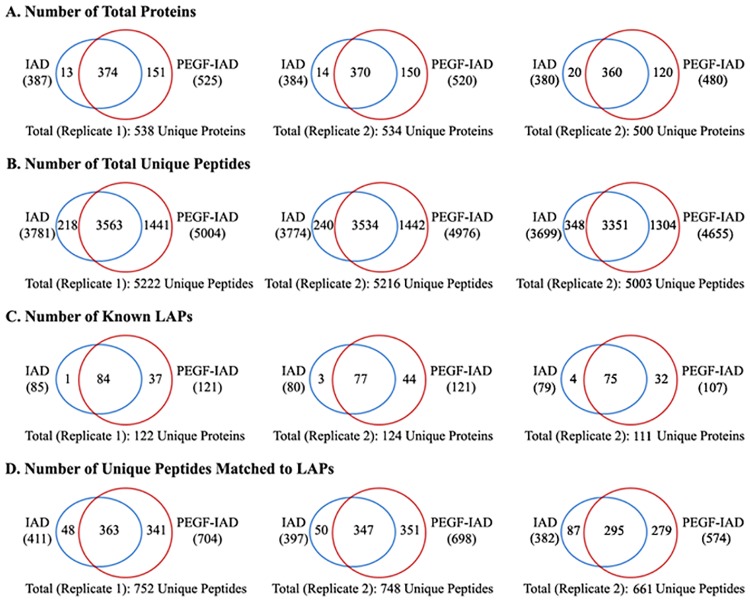
Comparison and distribution of the identified proteins including the known LAPs (A, C) and their matching unique peptides (B, D) using the conventional IAD method and the novel PEGF-IAD method developed in this study. In panels A–D, the replicate values are plotted separately to illustrate consistency in the overall trends.

To directly inspect and compare the differences resulting from inclusion of the PEGF step in plasma protein identification, the identified proteins and their matching unique peptides were separately analyzed in detail in three independent experiments. Fig 6 shows a side-by-side comparison of proteome coverage in the IAD and PEGF-IAD methods. We found that the numbers of proteins and unique peptides identified using the PEGF-IAD method were increased by 32.5±5.3% and 30.0±3.6% compared with that using the single IAD treatment, respectively, which were mainly reflect in the exclusively identified proteins and unique peptides.

The enhanced detection of LAPs achieved using the PEGF-IAD method is illustrated in detail in Fig 6. The Venn diagram displays the distribution of 135 known LAPs with concentrations below 100 ng/mL (based on previously published data) detected by LC-MS/MS in this study (S2 Table). In the three experiments, the PEGF-IAD method led to significant increases in the identification rate of known LAPs (by 43.0±7.9%) and corresponding unique peptides (by 65.8±13.6%). Comparatively, the increase in the extent of unique peptides identified was greater than that of the LAPs, potentially resulting in more reliable protein identification, as better protein identification by MS generally depends on a larger number of peptides. An increasing number of LAPs and unique peptides were identified exclusively with the PEGF-IAD method, while only a few proteins and unique peptides were exclusively identified by the IAD method. These results demonstrated that the PEGF-IAD method markedly facilitated the identification of plasma LAPs.

Between the three experiments, although all identified protein and peptide sets may have varied slightly, the overall trends in terms of performance were consistent. As reproducible sample processing steps are key requirements for quantitative applications in clinical proteomics, we initially evaluated the reproducibility of the PEGF-IAD method using Pearson plots of protein iBAQ values from three replicates (S2 Fig). The generated correlation coefficient values (R^2^) were consistent between both methods, which may be attributed to the individual reproducibility of the PEGF and IAD procedures. These observations therefore demonstrated good reproducibility of our integrated platform.

**Fig 7:**
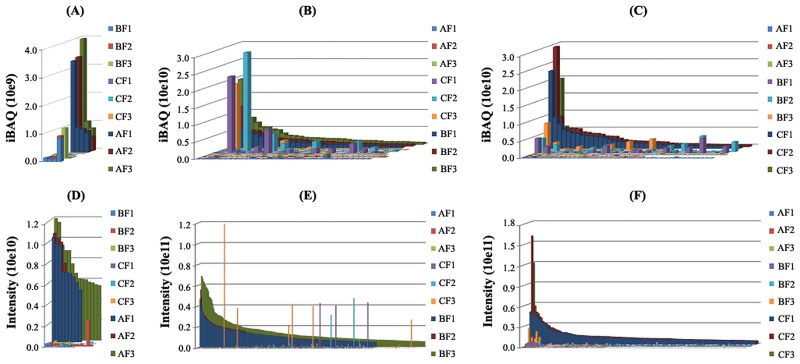
Comparison of MAPs (A, B, and C) and moderate-intensity peptides (D, E, F) identified from the three indicated fractions in replicate experiments, using the PEGF-IAD method. In panels A–F, the values for each replicate are plotted separately to illustrate consistency in the overall trends.

Besides the known LAPs, we also investigated a concrete manifestation of the moderate-abundance proteins and moderate-intensity peptides from LC-MS/MS analysis of the three fractions in the PEGF-IAD approach. For improved comparative-alignment analysis, proteins with iBAQ values of >5×10^8^ were defined as MAPs and peptides with intensity values of >5×10^9^ were defined as moderate-intensity peptides. As shown in Fig 7, both the MAPs and moderate-intensity peptides following the depletion of seven HAPs were efficiently separated. This effective separation indicates that including the PEGF step potentially lead to the observed improvements described herein.

## Discussion

In terms of the high complexity and large dynamic range of plasma components, comprehensive analysis of human plasma proteins is a challenging task. Hence, appropriate sample preparation, such as fractionation, is thus proposed before executing a deeper exploration for biomarker discovery [40]. The use of diverse fractionation techniques can remarkably raise the protein identifications [15]. To date, several different strategies based on HAP extraction or fractionation have been proposed in order to alleviate the signal-suppressive effects of HAPs by lowering the sample complexity and thereby improve the detection of LAPs [15, 41]. Despite not providing high resolution to fractionation the plasma sample relative to protein chromatography and isoelectric focusing, some precipitation methods relying on the use of common inorganic salts (ammonium sulfate) [42] and organic solvents (acetonitrile [43], TCA/acetone [44], ethanol [45], and PEG [36]) have been developed because of their simplicity and low cost to run. Alternatively, some chromatography-based separation and fractionation methods provide a degree of enrichment (or focusing) of a distinct subset of LAPs to improve the detection. Immunoaffinity chromatography with multiplexed antibodies, targetedly removing relatively abundant proteins, has been widely used for proteome profiling of human biofluids [46], even if potentially yielding protein loss due to nonspecific adsorption. In the LC format, immunoaffinity separations can effectively separate HAPs from relatively LAPs in a highly reproducible fashion, thus alleviating the HAP masking effects [2]. However, the results of one study demonstrated that while simplex IAD clearly enhanced the detection of high and moderate-abundance non-targeted proteins, it showed limited capacity for LAP detection [47]. Indeed, when a single-step analytical approach is used, the detection of LAPs proves challenging. Hence, various multi-step processes for penetrating deeper into the plasma proteome still need to be developed to achieve higher proteome coverage and thus enhance LAP detection [15].

In the present study, we aimed to integrate the commonly used precipitation approach with IAD. Thus, a precipitation method was designed as a sample fractionation procedure prior to IAD. Because effective immunoaffinity chromatography requires proteins to be in their native state, it is critical to select non-denaturing precipitating agents (e.g., ammonium sulfate, ethanol, and PEG) for compatibility with the downstream depletion step. Ammonium sulfate precipitation has been explored as a method for depleting some highly abundant proteins from blood plasma [42]. Ethanol precipitation has also been used to remove immunoglobulins and part of the albumin component prior to 2DE analysis [45]. In general, the formation and equilibration of precipitates take significantly less time with PEG compared to the time required when using ammonium sulfate or ethanol as a precipitating agent; therefore, PEG might be a preferred nonionic polymer for plasma protein fractionation [37]. Previously, PEG was used as a fractional precipitating agent only for protein purification, although its potential for the large-scale isolation of clinically useful proteins in human plasma had also been initially investigated [36]. By visual assessment, we found that the primary separation of the known, major protein components of plasma into individual fractions could be achieved using the PEG concentration described herein. Moreover, the ability of PEG to prevent plasma protein denaturation was further tested by non-denaturing electrophoresis. Indeed, we also deduced that PEG treatment did not interference with the downstream IAD step by inspecting the 2DE images. Our investigative results demonstrated that PEG enables the effective fractionation of plasma proteins and shows good compatibility with IAD. In light of these findings, we integrated PEGF with the classic IAD approach, aiming to reduce the plasma protein content complexity, as illustrated in Fig 1.

Through investigating the one-dimensional electrophoretic distribution of plasma proteins following differential PEG precipitation, we preliminarily determined that the Fb/Fn-contained fraction (A) can be obtained by 4%-PEG precipitation, the IgG-contained fraction (B) can be obtained by 12%-PEG precipitation, and albumin-contained fraction (C) can be obtained by 30%-PEG precipitation. Based on this fractionation trend, we attempted to visualize the detailed protein profile of the obtained three fractions post-IAD (AF, BF, and CF) by 2DE. Interestingly, the gel profiles showed that the protein spots in the BF and CF distributed differentially throughout the full range of MW and pI (Fig 3). The tendency observed in the 2DE images was comparable with that from iBAQ-based proteomic analysis. Based on the above analyses, we suggest that PEG precipitation is an effective corollary procedure for assigning post-IAD flow-through proteins and shows high potential for plasma proteome analysis.

The results from many studies have indicated that IAD has high reproducibility and offers an effective means for enhanced penetration into complex proteomes of human biofluid [2, 17, 41]. PEGF is a useful procedure that may in advertently introduce pre-analytical biased protein representation if the fractionation is not reproducible. From our initial evaluation after performing replicate analysis of three separate sample preparations, we found that the integrated PEGF-IAD platform showed good reproducibility that was comparable with the IAD method in terms of the protein iBAQ values. Consistency in protein identification of replicate analysis is necessary for the accurate determination of protein abundance changes in disease versus control clinical samples, especially for label-free quantitative proteomic analysis.

With our integrated PEGF-IAD strategy, although the IAD showed an inability to enhance global plasma protein identification, including the PEGF step conferred a noticeable advantage. When compared with the IAD method, we found that the integrated strategy could increase the overall plasma proteome coverage and peptide identification rate as evidenced by the LC-MS/MS results. Importantly, this enhanced capability enabled the identification of several LAPs, with concentrations in the ng/mL scope, including cytokines and growth factors from human subject. Comparatively, our integrated strategy resulted in increasing identification rate of known LAPs and matching unique peptides. Nearly all proteins and unique peptides, particularly known LAPs identified by the IAD method, were also identified by the PEGF-IAD strategy, as were a variety of additional proteins.

During LC-MS/MS analysis of plasma proteomes, tryptic digests of peptides derived from MAPs after the depletion of seven major HAPs could also obscure the detection of LAP products and thus mask their presence [47, 48]. In this study, we found that PEGF-IAD method produced greater peptide recovery and increased the detection of low-intensity peptides, thereby improving the identification efficiency of plasma proteins, particularly LAPs. The observed improvements in proteome coverage and LAP identification afforded by PEG were potentially attributable to the efficient separation of some moderately abundant proteins post-depletion of seven major HAPs, thereby alleviating severe signal suppression by their corresponding peptides in LC-MS/MS analysis. The enhanced detection of these low-level proteins is a significant step toward the discovery of potential candidate markers in plasma [49]. This is because indicators of a disease and/or healthy state such as tissue- and cell-specific proteins are typically present at lower concentrations (i.e., low ng/mL and pg/mL levels) in circulating blood plasma [50]. Therefore, the PEGF-IAD approach has the potential to aid in detecting LAPs of clinical interest.

In summary, we have described an enhanced, integrated strategy for analyzing the human plasma proteome that incorporates the ability of PEGF and IAD. The new integrated method is rapid and robust, and enabled improved proteome coverage and enhanced detection of LAPs. Thus, this combined method could aid in the discovery of plasma biomarkers of therapeutic and clinical interest. We anticipate that this method may also enhance the performance of multiple-reaction monitoring-based measurements for verification of biomarker candidates [51].

## Supporting Information

S1 Fig Plots of MW and pI versus iBAQ reciprocals of the plasma proteins identified from the BF and CF. In the two fractions, values for each replicate are plotted separately to illustrate consistency in the overall trends.
(TIF)

S2 Fig Reproducibility measurements of three replicates based on iBAQ values. Each point in the graph represents an identified protein, the X-axis shows iBAQ value for each protein tested in replicate 1 or 2, and the Y-axis shows the iBAQ value for each protein tested in replicate 2 or 3. The Pearson’s coefficient correlation values (R^2^) indicate the reproducibility of the PEGF-IAD platform.
(TIF)

S1 Table Analysis of human plasma proteins including known LAPs and their matching unique peptides identified from the 8 different samples analyzed in the present study. (XLSX)

S2 Table 135 known LAPs with reported concentrations below 100 ng/mL (based on previously published data) identified by LC-MS/MS in this study. (XLSX)
