# Supplementary figures and images for: Enhanced Detection of Low-Abundance Human Plasma Proteins by Integrating Polyethylene Glycol Fractionation and Immunoaffinity Depletion
Source: PLoS One. 2016 Nov 10;11(11):e0166306. doi: 10.1371/journal.pone.0166306 (PMC5104378; doi:10.1371/journal.pone.0166306)

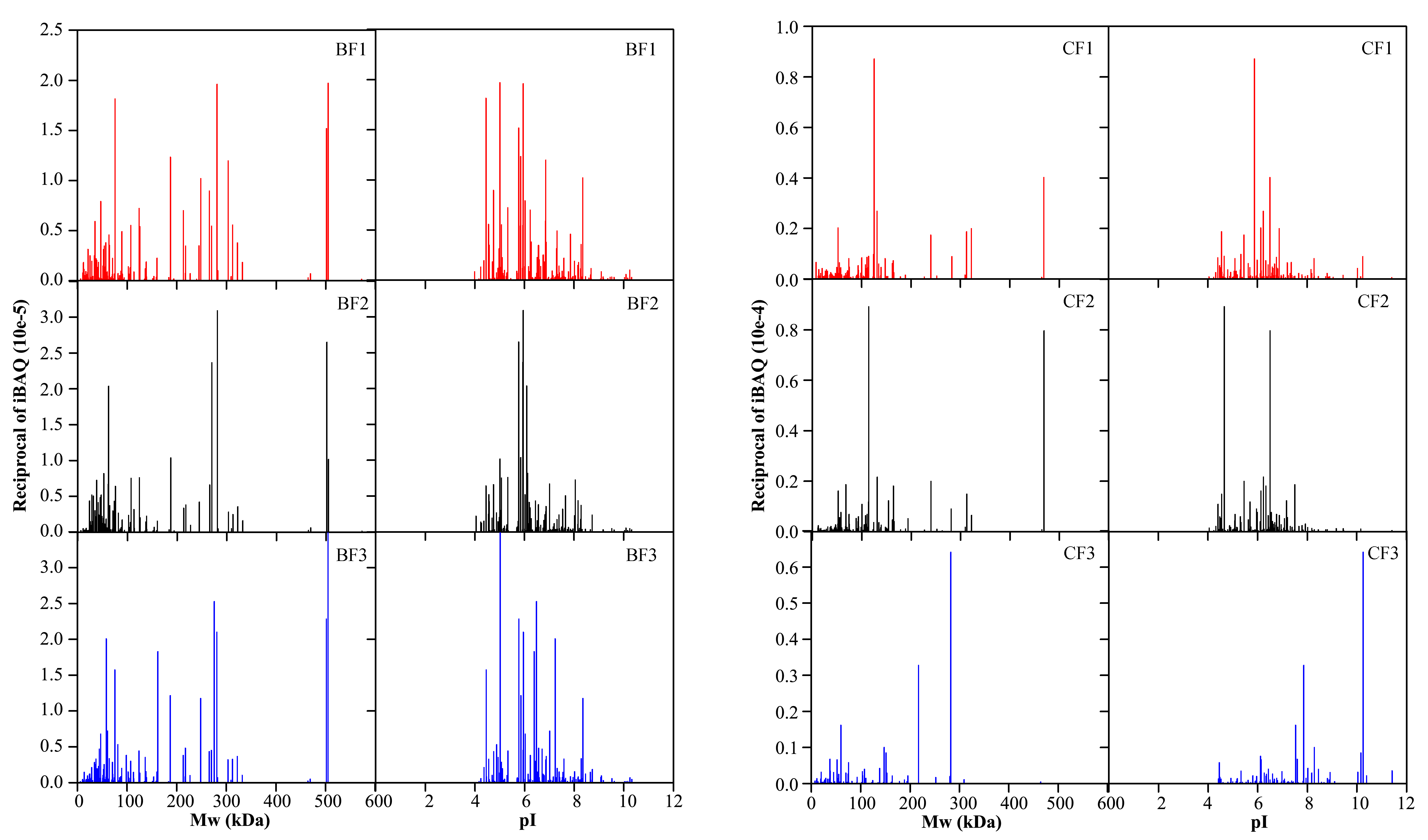

Supplement: S1 Fig — In the two fractions, values for each replicate are plotted separately to illustrate consistency in the overall trends. (TIF) [file pone.0166306.s001.tif]

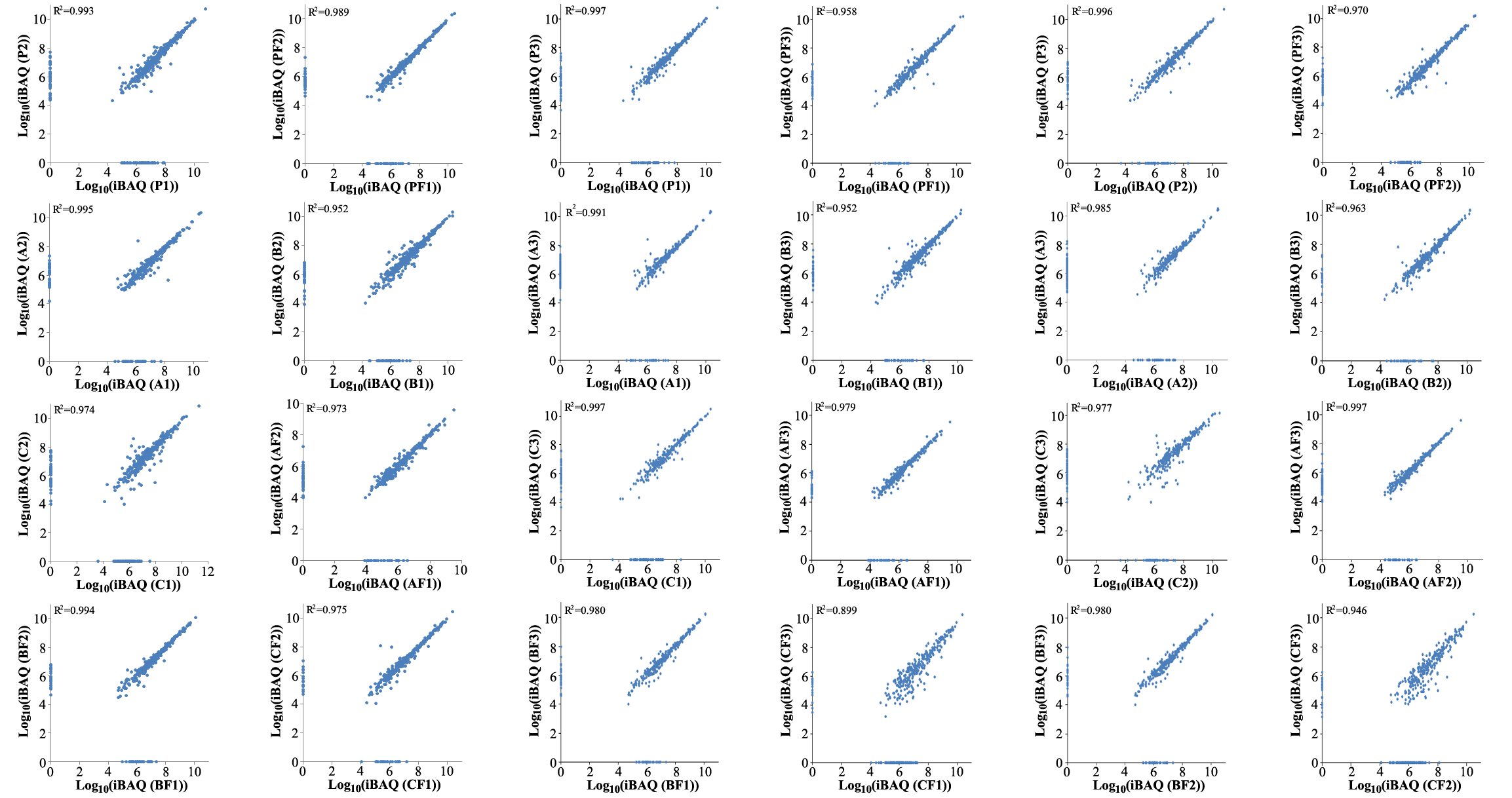

Supplement: S2 Fig — Each point in the graph represents an identified protein, the X-axis shows iBAQ value for each protein tested in replicate 1 or 2, and the Y-axis shows the iBAQ value for each protein tested in replicate 2 or 3. The Pearson’s coefficient correlation values (R2) indicate the reproducibility of the PEGF-IAD platform. (TIF) [file pone.0166306.s002.tif]
